# Income Volatility and Depressive Symptoms among Elderly Koreans

**DOI:** 10.3390/ijerph16193580

**Published:** 2019-09-25

**Authors:** Sujin Kim, S.V. Subramanian

**Affiliations:** 1 Department of Health Care Policy Research, Korea Institute for Health and Social Affairs, Sejong 30147, Korea; 2 Institute of Health and Environment, Seoul National University, 1 Gwanak-ro, Gwanak-gu, Seoul 08826, Korea; 3 Department of Social and Behavioral Sciences, Harvard T.H. Chan School of Public Health, Boston, MA 02115, USA; svsubram@hsph.harvard.edu; 4 Harvard Center for Population and Development Studies, 9 Bow Street, Cambridge, MA 02138, USA

**Keywords:** income volatility, depression, older adults, living arrangement, South Korea

## Abstract

This study examines the relationship between volatile income and depression, and moderating effects of living arrangements among older adults in South Korea. Using the Korean Longitudinal Study of Aging, we studied 4123 adults aged 60 or older. Income volatility was defined as the variance of logged income across four assessments from 2006 to 2012. Depression was measured as the Center for Epidemiologic Studies Depression (CES-D) scores in 2012. It was examined whether income volatility was related to depressive symptoms, and whether the association depended on co-residence with children. In results, income volatility was not related to CES-D scores in main-effect models without an interaction term. The relationship between income volatility and depressive symptoms depended on co-residence with children (*p* < 0.001). Higher income volatility was linked to increased risks of CES-D scores among the elderly living without children (incident rate ratio (IRR): 1.27, 95% confidence interval (CI): 1.07–1.50, *p*-value: 0.005) whereas it was related to lower CES-D scores among those co-residing with children (IRR: 0.68, 95% CI: 0.52–0.88, *p*-value: 0.003). Absolute income volatility has detrimental psychological consequences for older adults who live on their own. The finding implies that social protection policies for elderly households that live with an unstable income are needed.

## 1. Introduction

The elderly are retired, and have limited chances for paid work. In societies with limited safety nets, elder’s limited productivity as well as job loss could result in poverty and unstable income. For example, increased income volatility as well as downward income mobility are often observed in the older population [1,2]. Considering the growing old-age population and lack of social security systems in many countries [3,4], there is a clear need to better understand how low income and unstable income affect elderly well-being. Indeed, cumulative evidence has shown the negative health effects of low income and poverty [5,6,7,8]. However, little attention has been given to the health consequences of fluctuating and volatile income. 

Income volatility is generally considered to be a rapid and unpredictable change in income over time and is measured in various ways; e.g., a continuous value of the sum of the squared deviations of each logged income from their mean logged income [9,10]; tertile for the intra-individual standard deviation of the percent change in income [11]. It is a concern that income volatility is often accompanied by income uncertainty, which could become a source of chronic worry [12]. Income uncertainty may prohibit an individual from paying for current needs, as well as hinder future planning [13,14]. These could lead an individual to suffer a higher level of stress and depression due to financial insecurity [8]. This would be applicable especially to elderly people, who are not only more like to have volatile income but also to be vulnerable psychologically to income fluctuation [11]. Nevertheless, volatile income might not be harmful. Income change may be predictable, and shocks due to the change could be smoothed [14]. This would occur especially if income shocks could be smoothed and did not prevent them from making a living. In this regard, the existence of social security systems or other social arrangements, which cover the basic needs of the elderly, could reduce or eliminate disadvantages due to unstable income. Indeed, family support through co-residence still plays an important role in elderly financial wellbeing, especially in countries where the safety net for the elderly is under-developed [15,16], and this social arrangement might moderate the inimical effects of volatile income on depression by providing a kind of social security for the elderly. South Korea (hereafter, Korea) presents a particularly interesting context in which to examine these associations. In Korea, co-residence with adult child is an important factor in reducing the risk of poverty among older adults [16], although support from children has continuously decreased. Since a national pension system was introduced as a contributory pension in 1988, only about 30% of adults age 65 or older received the benefits in 2012 [17]. These result in high elderly poverty rate; e.g., approximately half of them live below the relative poverty line [18]. Moreover, their psychological health remains at great risk. The suicide rate in the population aged 65 and older in Korea more than doubled in 10 years, rising from 34 in 2000 to 72 deaths in 2010 (per 100,000) [19], and stood at 70 deaths for the population aged 80 and older in 2017 [20]. Although financial hardship is regarded as an important factor, few studies investigated the effects of unstable economic conditions on health among older adults in Korea.

In this regard, it would be hypothesized that income volatility has a harmful impact on psychological health among older adults in South Korea, especially elderly household who live without children could be more vulnerable for income volatility than those co-residing with children. Thus, the aim of this study is to examine the relationship between income volatility and depressive symptoms among older adults in South Korea with underdeveloped old-age safety nets and family-oriented welfare system. By using longitudinal data of older adults from the Korean Longitudinal Study of Aging (KLoSA) that contains repeated observations on the same individuals from 2006 to 2012, the study seeks to: (1) assess whether income volatility that is measured with couple-level income over the past six years is related to depressive symptoms; (2) determine whether a social arrangement such as co-residing with children moderates the effects of income volatility on depressive symptoms among older Koreans.

## 2. Materials and Methods

### 2.1. Data and Study Population

The data for this study were derived from KLoSA. Similar to the Health and Retirement Study (HRS) in the United States and the English Longitudinal Study of Ageing (ELSA) in England, KLoSA is a nationally representative longitudinal study on middle- and old-aged community-dwelling adults (aged 45 years or older) in South Korea, which is administered by the Korea Employment Information Service (KEIS). The study selected 1000 enumeration districts (EDs) based on area (urban/rural) and type of housing (apartment/ordinary housing) and then six households within each ED, by using a multistage stratified area probability sampling design. It was first conducted in 2006 for 10,254 individuals of 6171 households, and has had biennial follow-up waves since then. The survey is conducted by skilled interviewers in the participants’ homes using a structured questionnaire, which consists of seven main sections: demographic characteristics, household and family characteristics, health status, employment, income, assets, and subjective expectations and satisfaction. More detailed information on the survey approach can be found at the KLoSA website (http://survey.keis.or.kr/ENCOMAM0000N.do) [21]. This study is secondary analysis of publicly available de-identified data, which is exempt from Institutional Review Board approval.

To measure income volatility, this study used participants that completed the first four waves of the survey, from 2006 to 2012. The follow-up rates were 88.0%, 80.3% and 76.2% for the 2008, 2010 and 2012 waves, respectively [22]. Our analytic sample includes all adults age 60 or older as of 2012 that completed four waves of the study (*N* = 4123).

### 2.2. Outcome Measure

Depressive symptoms were measured by using the short-form Center for Epidemiologic Studies-Depression 10-item Scale (CES-D10) developed at the Boston site of Established Populations for Epidemiological Studies of the Elderly, The CES-D 10 is a brief screening instrument to assess depressive symptoms in older adults [23,24]. It asks about the presence of the following features over the previous week: “I felt depressed”, “I felt that everything I did was an effort”, “My sleep was restless”, “I was happy”, “I felt lonely”, “People were unfriendly”, “I enjoyed life”, “I felt sad”, “I felt that people disliked me” and “I could not get going” [23]. In KLoSA, the response for each item ranges from 0 (very rarely: less than one day) to 3 scale (almost always: five to seven days). As the original Boston version of the CES-D 10 has two response options (yes, no) [23], the scales of 1–3 were coded as 1 (yes) [25,26], and then the scores for all were summed into a composite score ranging from 0–10 [23].

### 2.3. Income Level and Income Volatility 

The key explanatory variable of the study is income volatility, which was measured based on income of each couple at all four waves. Since the KLoSA has been administered every even-numbered year since 2006, the monthly income of each respondent in the 2006, 2008, 2010, and 2012 survey was calculated including earnings, asset income, and public and private transfers. A couple’s income was defined as the sum of respondent’s and spouse’s income, which were deflated with the consumer price index using 2006 as the base year [27]. Absolute income volatility was measured as the transitory component of income; that is, the variance of logged income over the four waves was calculated as the sum of the squared deviations of each logged income from their mean logged income [9,10]. The mean of income (divided by the square root of 2 for the married) at the four waves was calculated for the variable of income level. Income level and income volatility were used as continuous variables with log transformation.

### 2.4. Covariates 

Possible covariates are considered. Age, sex, marital status (married, unmarried), education (elementary, middle, high schools, ≥college), current occupation (not working, employee, self-employed, unpaid family workers), residence of living (metropolitan, city, rural), and co-residence with children (yes, no) were collected. Physical health was assessed with three variables. The number of chronic diseases was categorized into three groups (0, 1, and 2 or more), based on the following self-reported medical conditions diagnosed by a physician: hypertension, diabetes, cancer, lung disease, heart problems, stroke, arthritis, and gastrointestinal disease. Physical function was measured using two scales, both activities of daily living (ADL) and instrumental activities of daily living (IADL). ADL scale included the ability to dress, wash (face/teeth/hair), bathe, eat, get out of bed, use restroom, and control urination. IADL scale includes personal grooming, going out for short distances, using transportation, making/receiving phone calls, managing money, doing household chores, preparing meals, shopping, taking medications, and doing the laundry. ADL and IADL were respectively dichotomized into 0 (no dependency) and 1 (dependent in one or more activities). In addition, baseline CES-D score was included. 

Multicollinearity was assessed based on both bivariate correlations and variance inflation factor (VIF) because this makes some variables statistically insignificant by increasing standard errors of the coefficients. All of the bivariate correlations were less than 0.50 (Table S1). In addition, the variance inflation factors for the variables were all less than 2.0, which indicate that multicollinearity does not cause problems in the regression models (data not shown) [28].

### 2.5. Estimation Strategy

To fill the void of research on income volatility and depression, this study tests two hypotheses. The first predicts that couples who experience more absolute volatility in their income will report higher levels of depression. The second hypothesis predicts that living with children buffers the harmful effects of income volatility on depression. Potential confounders including baseline depression that may cause reverse causation were adjusted for. 

To identify a relationship between income volatility and elderly depression, we specify our main model as follows:

(1) $$y_{i}=\beta_{0}+\beta_{1}income\;volatility_{i}+\beta_{2}co-residence_{i}+\beta_{3}x_{i}+\varepsilon_{i},$$

where *y* is CES-D score; income volatility is the variance of couple income; co-residence is whether an individual lives with his/her child; *x* denotes other individual factors; and *ε_i_* is a random error. 

We expect that the effects of income volatility depend on co-residence with children, and added the interaction term of income volatility and co-residence.

(2) $$y_{i}=\beta_{0}+\beta_{1}income\;volatility_{i}+\beta_{2}co-residence_{i}+\beta_{3}income\;volatility\cdot co-residence_{i}+\beta_{4}x_{i}+\varepsilon_{i},$$

Negative binomial regression was used to model the dependent variable that represents counts [29,30,31]. Whereas count data are often modeled using Poisson regression, negative binomial regression is more appropriate for the data with a large variation because Poisson distribution is assumed to have variance equal to mean. For all analyses, we used Stata ver 12.0/SE, and set the level of significance as 0.05 (two-sided), estimating robust standard errors. 

## 3. Results

### 3.1. Descriptive Characteristics

Table 1 presented the characteristics of the study population. Among the total of 4123 subjects, 33.2% live with children. Men than women (45.0% vs. 39.0%) and respondents without a spouse than the married (25.0% vs. 39.1%) are more likely to co-reside with children. In the elderly living without children, income volatility is slightly lower (0.09 vs. 0.12) and the not-working is lower (69.2% vs. 74.9%). Income, net asset, educational level, number of chronic disease and baseline depression are not very different in two groups.

### 3.2. Relationships of Income Volatility to Depressive Symptoms 

Table 2 displayed the results from the multiple regression models. First, we assessed the relation of income volatility measured over the past six years to depressive symptoms in the full sample and for each gender, as seen in models 1–3. Overall, there was no statistically significant evidence that income volatility was associated with increased/decreased depressive symptoms while higher income level was related to fewer depressive symptoms, particularly in women. In addition, co-residence with children is not related to depression. Poorer health, such as more chronic disease, and greater difficulties in IADL and ADL, is associated with higher CES-D scores. 

Since co-residing with children could give financial advantages to the elderly [16], the linkage of income volatility to depression in the elderly may differ if they live with children. We added an interaction term of income volatility and co-residence with children to the models, which are shown in models 4–6. The interaction term shows a significant coefficient, which means the relation of income volatility to depression depends on the living arrangement. Among individuals not co-residing with children, more volatile income is related to higher depressive symptoms. The need for the interaction term was evaluated using the Wald test [29], which showed statistical significances in the full sample, and in men and women. 

**Figure 1:**
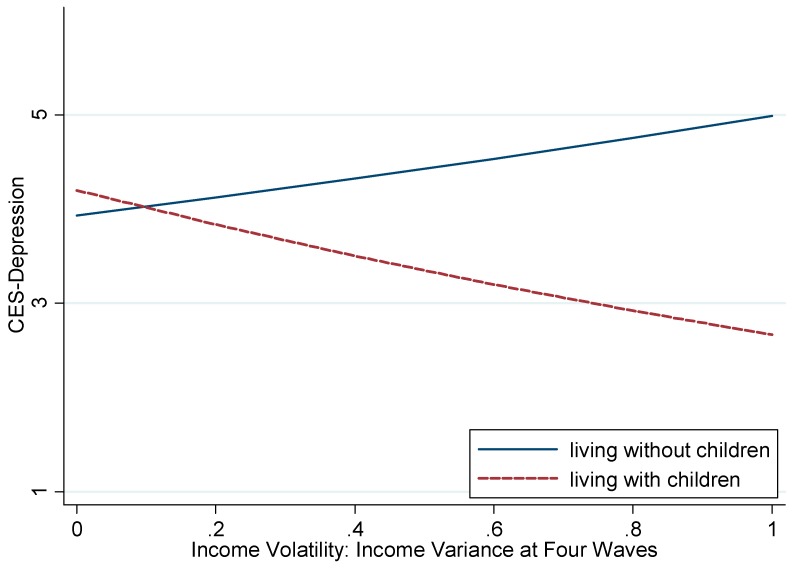
The predicted Center for Epidemiologic Studies-Depression (CES-D) 10 scores across income volatility by co-residence with children in full sample. Significantly different relationship between income volatility and CES-D 10 scores by living with children (*p*-value of interaction term (between income volatility and living with children) <0.001, *p*-value of Wald test <0.001).

The predicted depression scores were estimated from models 4–6 of Table 2. Figure 1 shows the distribution of depression across income volatility by co-residence. In the full sample, income volatility was positively related to depressive symptoms among individuals living without children. That is, the more volatile income people have, the more depressive symptoms they have (incident rate ratio (IRR): 1.27, 95% confidence interval (CI): 1.07–1.50, *p*-value: 0.005). Surprisingly, among older adults who lived with children, absolute volatility showed a protective association with depression, which was not anticipated; the more volatile income people have, the fewer depressive symptoms they have (IRR: 0.68, 95% CI: 0.52–0.88, *p*-value: 0.003).

We conducted sensitivity analyses to assess the robustness of the results. A potential concern is heterogeneity between the two groups. We split the sample by co-residence with children and performed analyses for each sample. These analyses yielded essentially the same results (see Models 1–2 of Table S2). For example, in the elderly co-residing with their children, a 10% increase in income volatility is approximately related to a 2.3% decrease in depression scores (IRR: 1.25, 95% CI: 1.05–1.48 for without children; IRR: 0.66, 95% CI: 0.49–0.87 for with children), similar to the result obtained in the model using interaction term. We carried out sensitivity analyses by including additional covariates such as household income and household wealth in models, which did not change the findings (see Models 3–4 of Table S2).

## 4. Discussion

The present study examined the relation of income volatility to depressive symptoms among older adults in Korea. The analysis showed no significant relationship between income volatility and depressive symptoms in the main effect model; however, the model with the interaction term of absolute income volatility and co-residence with children showed the relationship depended on living arrangements. Higher income volatility was linked to increased risk of depression among older adults living without a child, whereas it was related to fewer depressive symptoms among those co-residing with a child. This finding suggests the potential detrimental consequences of income uncertainty for psychological health among older adults living alone or with a spouse.

The findings are partially consistent with our expectation of a harmful relation of income volatility to depressive symptoms. Along with a decline in work-related payments that was a definite regular income source, the elderly would live with fluctuating and volatile income in the absence of other income support systems [2,5,32]. Income fluctuation could not only hamper planning for the future but also prohibit older adults from purchasing necessities to meet current needs, such as food consumption [12,33]. In particular, income shocks experienced by the elderly are possibly chronic rather than temporal. Income insecurity and continuous financial strain may lead the elderly to suffer higher levels of distress, and consequently to be depressed. This may be especially true for old-age people who live alone or with a spouse. They are likely unable to buffer such income shocks or do have limited resources to smooth income fluctuations. As a result, the detrimental health consequences of volatile income would be significant in elderly household. Indeed, our results show that income volatility by itself may contribute to higher depressive symptoms in elderly households, but not the elderly living with children.

Previous literature that focuses on younger adults showed the harmful impact of income volatility, and especially downward income mobility, on health. A 15-year follow-up cohort of young adults in the US found that income volatility and drops were associated with a nearly 2-fold risk of cardiovascular disease and all-cause mortality [11] while another study for young age group in the US found that the frequency of significant downward volatility was associated with depressive symptoms, but not volatile income by itself [12]. While few studies examined the association between volatile income and health among older adults, a longitudinal study for U.S. adults older than 50 years found a loss of net worth was related to an increased risk of all-cause mortality [34].

In general, extended living arrangements help buffer the effects of labor market disadvantages faced by individual household members [35,36]. Similarly, to the elderly, co-residence with economically active members in the same household may alleviate not only the risk of poverty but also the harmful effects of income volatility [15,16]. Nevertheless, it is unexpected that more volatile income was related to fewer depressive symptoms in the elderly living with children. A possible explanation is that older adults may prefer their exit from onerous work and increase in leisure time, working flexibly [37]. Even if their income becomes volatile, co-residence with children would secure their basic livelihood. For example, a study found retirement is beneficial to health through causing relief from work-related stress and strain, an increase in sleep duration and an increase in physical activity [38]. 

In the present study, co-residence with children did not affect depressive symptoms among older adults. It might be expected living with a child is beneficial to older adults in a non-western context where an intergenerational living arrangement is common. The associations, however, are inconsistent. One study in rural China reported that although it was associated with a higher quality of life in short and mid-terms, living with adult children increased the mortality risk of cardiovascular disease [39]. Another study on adult child migration in Moldova found that the migration did not affect depression and did increase self-reported health among elderly parents, which may be related to increases in remittances despite decreased social contact with their migrant family members [40]. A study in the U.S. found older adults living alone had lower odds of reporting poor/fair health than those living with others [41]. Another study in the U.S. found that older married couples may not benefit from the emotional or instrumental support provided by their children due to lower self-esteem [42]. 

This study has limitations. First, participants who are missing income data at interviews must be excluded. In the present study, such attrition seemed to be related to the exclusion of those with higher volatility and fewer resources. If these participants felt greater impacts of volatility, the present findings would give a conservative estimate of the negative effect. Second, intergenerational co-residence is determined based on preference of both parents and children, and parents’ physical and mental health. Although observed characteristics were not very different between two groups, and a wide range of variables including prior depressive symptoms were adjusted for, the two elderly groups might have systematically different features. Lastly, very different events may increase income volatility. Some of them may be predictable on the one hand; some of them may not be predictable on the other hand. These different characteristic may have different effects on psychological health. It is not possible, however, to distinguish them in the present study.

## 5. Conclusions

Our results suggest that absolute income volatility has detrimental health consequences in elderly households, and that a social arrangement such as co-residence with children moderates such negative effects. The finding implies that social protection policies for elderly households that live with an unstable income are needed in South Korea. In addition, it is unexpected that higher absolute income volatility is linked to fewer depressive symptoms in the elderly living with children. Further investigations are needed to fully understand the mechanism and public health consequences of these findings. In addition, it would be required to explore whether these patterns are observed in other contexts.

## Figures and Tables

**Table 1:** General characteristics of study population.

Characteristic ^a^	All		Living without Children		Living with Children		
	% or Mean	SD	% or Mean	SD	% or Mean	SD	*p*-value
No. of observations	4123	-	2753	-	1370	-	
CES-D 10 score, mean (SD)	4.00	3.00	3.96	2.97	4.07	3.04	0.27
Male, %	43.0%	-	45.0%	-	39.0%	-	<0.001
Age (years), mean (SD)	71.79	7.80	71.55	7.19	72.26	8.89	0.006
Without a spouse, %	29.7%	-	25.0%	-	39.1%	-	<0.001
Income level ^b^, mean (SD)	88.14	80.18	89.81	76.81	84.79	86.48	0.06
Income volatility, mean (SD)	0.10	0.15	0.09	0.14	0.12	0.16	<0.001
Net asset ^b^, mean (SD)	20,968	35,649	21,118	31,883	20,667	42,225	0.70
Current working status							<0.001
Not working, %	71.1%	-	69.2%	-	74.9%	-	
Employee, %	10.1%	-	9.3%	-	11.8%	-	
Self-employed, %	14.4%	-	16.0%	-	11.2%	-	
Unpaid family worker, %	4.4%	-	5.5%	-	2.1%	-	
Education							0.64
≤Elementary, %	28.1%	-	27.6%	-	29.1%	-	
≤Middle School, %	32.6%	-	33.2%	-	31.3%	-	
≤High school, %	15.4%	-	15.4%	-	15.6%	-	
≥College, %	23.9%	-	23.8%	-	24.1%	-	
Place of living							<0.001
Metropolitan, %	39.4%	-	35.6%	-	47.1%	-	
City, %	29.9%	-	28.3%	-	33.3%	-	
Rural, %	30.6%	-	36.1%	-	19.6%	-	
No. of Chronic disease							0.40
0, %	26.6%	-	26.0%	-	27.8%	-	
1, %	33.1%	-	33.1%	-	33.0%	-	
2+, %	40.4%	-	40.9%	-	39.2%	-	
IADL 1+, %	15.6%	-	13.4%	-	19.9%	-	<0.001
ADL 1+, %	6.5%	-	5.3%	-	9.1%	-	<0.001
Baseline CES-D 10 score, mean (SD)	3.18	2.71	3.23	2.73	3.09	2.66	0.102

Abbreviations: CES-D 10, Center for Epidemiologic Studies-Depression 10-item Scale; IADL, instrumental activities of daily living; ADL, activities of daily living; SD, standard deviation; ^a^ All variables were measured in 2012, except for baseline CES-D 10 score measured in 2006, and income level and volatility; ^b^ The unit is 10,000 Korean won, which is approximately equivalent to 10 USD.

**Table 2:** The relationships of income volatility to depressive symptoms (CES-D 10 scores).

	(1) Main Effect	(2) Main Effect	(3) Main Effect	(4) Interaction Effect	(5) Interaction Effect	(6) Interaction Effect
	All	Men	Women	All	Men	Women
	ß (95% CI)	ß (95% CI)	ß (95% CI)	ß (95% CI)	ß (95% CI)	ß (95% CI)
Co-residence	−0.01	0.05	−0.03	0.07 *	0.14 **	0.03
	(−0.06–0.04)	(−0.03–0.13)	(−0.09–0.03)	(0.01–0.13)	(0.04–0.24)	(−0.04–0.11)
Income volatility	0.004	0.03	−0.03	0.24 **	0.29 *	0.18
	(−0.14–0.15)	(−0.21–0.26)	(−0.22–0.16)	(0.07–0.40)	(0.04–0.55)	(−0.05–0.40)
Co-residence	-	-	-	−0.69 ***	−0.87 **	−0.55 **
income volatility				(−1.02–−0.36)	(−1.41–−0.32)	(−0.96–−0.15)
Male	0.01	-	-	0.01	-	-
	(−0.005–0.02)			(−0.004–0.02)		
Age	0.004 *	0.01 *	0.004	0.004 *	0.01 *	0.004
	(0.003–0.01)	(0.0001–0.01)	(−0.001–0.01)	(0.0003–0.01)	(0.00004–0.01)	(−0.002–0.01)
Without a spouse (ref: with a spouse)	0.06 *	0.16 **	0.04	0.06 *	0.16 **	0.04
	(0.01–0.12)	(0.04–0.27)	(−0.03–0.11)	(0.01–0.12)	(0.04–0.27)	(−0.02–0.11)
Income level	−0.06 ***	−0.05 ^†^	−0.08 ***	−0.07 ***	−0.05 ^†^	−0.07 ***
	(−0.10–−0.03)	(−0.10–0.003)	(−0.12–−0.03)	(−0.10–−0.03)	(−0.11–0.0001)	(−0.12–−0.03)
Net asset	0.06	0.05	0.09	0.06	0.05	0.09
	(−0.06–0.18)	(−0.13–0.22)	(−0.06–0.25)	(−0.06–0.17)	(−0.13–0.22)	(−0.06–0.25)
Employee (ref: no working)	−0.21 ***	−0.17 *	−0.26 ***	−0.21 ***	−0.17 *	−0.26 ***
	(−0.31–−0.11)	(−0.31–−0.03)	(−0.41–−0.11)	(−0.31–−0.11)	(−0.31–−0.03)	(−0.41–−0.10)
Self-employed (ref: no working)	−0.12 **	−0.22 ***	0.11 ^†^	−0.12 **	−0.22 ***	0.11 ^†^
	(−0.20–−0.04)	(−0.32–−0.11)	(−0.01–0.23)	(−0.20–−0.05)	(−0.33–−0.12)	(−0.01–0.23)
Unpaid family worker (ref: no working)	−0.24 ***	−0.27 *	−0.24 ***	−0.24 ***	−0.26 ^†^	−0.24 ***
	(−0.36–−0.12)	(−0.54–−0.01)	(−0.38–−0.11)	(−0.36–−0.12)	(−0.54–0.01)	(−0.38–−0.11)
≤Elementary (ref: ≥College)	0.01	0.02	0.01	0.01	0.01	0.01
	(−0.07–0.09)	(−0.11–0.14)	(−0.11–0.12)	(−0.07–0.09)	(−0.11–0.13)	(−0.11–0.13)
Middle school (ref: ≥College)	0.02	0.02	0.01	0.02	0.01	0.01
	(−0.05–0.09)	(−0.08–0.12)	(−0.10–0.12)	(−0.05–0.09)	(−0.09–0.11)	(−0.10–0.12)
High school (ref: ≥College)	0.004	0.01	−0.01	−0.0001	0.004	−0.01
	(−0.08–0.09)	(−0.10–0.12)	(−0.14–0.12)	(−0.08–0.08)	(−0.11–0.12)	(−0.14–0.12)
City (ref: Metropolitan)	0.08 **	0.12 *	0.07 ^†^	0.08 **	0.12 *	0.06 ^†^
	(0.03–0.14)	(0.03–0.21)	(−0.001–0.14)	(0.03–0.14)	(0.03–0.21)	(−0.004–0.13)
Rural (ref: Metropolitan)	0.03	0.06	0.02	0.03	0.07	0.02
	(−0.02–0.09)	(−0.03–0.16)	(−0.05–0.09)	(−0.02–0.09)	(−0.03–0.17)	(−0.05–0.09)
Chronic disease: 1 (ref: none)	0.05	0.04	0.07	0.05	0.03	0.07
	(−0.01–0.12)	(−0.06–0.14)	(−0.02–0.15)	(−0.01–0.12)	(−0.06–0.13)	(−0.01–0.15)
Chronic disease: 2+ (ref: none)	0.14 ***	0.09 ^†^	0.18 ***	0.14 ***	0.09 ^†^	0.18 ***
	(0.08–0.20)	(−0.01–0.19)	(0.10–0.26)	(0.08–0.20)	(−0.004–0.19)	(0.11–0.26)
IADL: 1+	0.22 ***	0.29 ***	0.17 ***	0.22 ***	0.28 ***	0.17 ***
	(0.16–0.29)	(0.18–0.40)	(0.09–0.26)	(0.16–0.29)	(0.17–0.39)	(0.09–0.26)
ADL: 1+	0.15 ***	0.15 *	0.15 **	0.14 ***	0.15 *	0.15 **
	(0.06–0.23)	(0.02–0.28)	(0.05–0.26)	(0.06–0.22)	(0.02–0.27)	(0.05–0.25)
2006 CES-D 10	0.07 ***	0.08 ***	0.06 ***	0.07 ***	0.08 ***	0.06 ***
	(0.06–0.07)	(0.06–0.09)	(0.05–0.07)	(0.06–0.07)	(0.06–0.09)	(0.05–0.07)
Wald test *p*-value				<0.001	0.008	0.041
No. of observations	4123	1772	2351	4123	1772	2351

Abbreviations: CES-D 10, Center for Epidemiologic Studies-Depression 10-item Scale; IADL, instrumental activities of daily living; ADL, activities of daily living; CI, confidence interval; *** *p* < 0.001, ** *p* < 0.01, * *p* < 0.05, ^†^ *p* < 0.1; Negative Binomial regressions on CES-D 10 score from the 2012 KLoSA (robust standard errors); all adjusted variable, except 2006 CES-D 10 and income level and volatility, are collected from the 2012 KLoSA.

## Supplementary Materials

The following are available online at https://www.mdpi.com/1660-4601/16/19/3580/s1, Table S1: Bivariate correlation, Table S2: The relationships of income volatility to depressive symptoms (sensitivity analysis)
