# Supplementary material for: Income Volatility and Depressive Symptoms among Elderly Koreans
Source: Int J Environ Res Public Health. 2019 Sep 25;16(19):3580. doi: 10.3390/ijerph16193580 (PMC6801608; doi:10.3390/ijerph16193580)
Supplement: Supplementary file 1 [file ijerph-16-03580-s001.pdf]

Supplementary Information

Supplementary Table 1. Bivariate correlation.

	**1**	**2**	**3**	**4**	**5**	**6**	**7**	**8**	**9**	**10**	**11**	**12**	**13**	**14**	**15**
1. CES-D	1.00														
2. CES-D (2006)	0.33	1.00													
3. Income level	−0.25	−0.28	1.00												
4. With children	0.01	−0.02	−0.07	1.00											
5. Income volatility	0.04	0.04	−0.14	0.06	1.00										
6. Gender	0.11	0.16	−0.23	0.05	0.03	1.00									
7. Age	0.24	0.20	−0.46	0.04	0.04	0.08	1.00								
8. With spouse	0.18	0.22	−0.40	0.14	0.06	0.39	0.37	1.00							
9. Job	−0.18	−0.12	0.28	−0.08	−0.09	−0.16	−0.26	−0.20	1.00						
10. Education	−0.18	−0.29	0.46	0.00	0.01	−0.41	−0.38	−0.34	0.06	1.00					
11. Chronic disease	0.18	0.17	−0.15	−0.02	0.00	0.08	0.18	0.10	−0.16	−0.13	1.00				
12. IADL	0.25	0.14	−0.18	0.09	0.02	−0.05	0.31	0.10	−0.16	−0.11	0.13	1.00			
13. ADL	0.23	0.15	−0.16	0.07	0.04	0.01	0.28	0.10	−0.14	−0.13	0.10	0.59	1.00		
14. Residence	0.01	0.05	−0.08	−0.16	−0.01	0.00	0.05	−0.02	0.26	−0.21	−0.04	−0.07	−0.04	1.00	
15. Wealth	−0.12	−0.17	0.37	−0.03	−0.02	−0.13	−0.22	−0.29	0.12	0.27	−0.06	−0.14	−0.11	0.01	1.00

Supplementary Table 2. The relationships of income volatility to depressive symptoms (sensitivity analysis).

	(1)	(2)	(3)	(4)
	**CES-D 10**	**CES-D 10**	**CES-D 10**	**CES-D 10**
	**Living without Children**	**Living with Children**	**All**	**All**
	**ß (95% CI)**	**ß (95% CI)**	**ß (95% CI)**	**ß (95% CI)**
Co-residence	-	-	−0.00	0.07 *
			(−0.06–0.05)	(0.01–0.14)
Income volatility	0.22 *	−0.42 **	0.01	0.25 **
	(0.05–0.39)	(−0.71–−0.14)	(−0.14–0.16)	(0.09–0.42)
Co-residence · income volatility	-	-	-	−0.74 ***
				(−1.08–−0.41)
Male	0.02 *	−0.00	0.01	0.01
	(0.00–0.04)	(−0.03–0.02)	(−0.01–0.02)	(−0.01–0.02)
Age	0.01 **	0.00	0.00	0.00
	(0.00–0.01)	(−0.01–0.01)	(−0.00–0.01)	(−0.00–0.01)
Single (married)	0.09 **	0.03	0.06 *	0.06 *
	(0.03–0.16)	(−0.08–0.13)	(0.00–0.12)	(0.00–0.12)
Income level	−0.08 ***	−0.05	−0.05 **	−0.05 **
	(−0.12–−0.04)	(−0.11–0.01)	(−0.09–−0.02)	(−0.09–−0.02)
Net asset	0.13 †	−0.04	−0.13	−0.13
	(−0.02–0.28)	(−0.23–0.14)	(−0.33–0.07)	(−0.33–0.06)
No working	1.00	1.00	1.00	1.00
	[reference]	[reference]	[reference]	[reference]
Employee	−0.21 **	−0.21 *	−0.21 ***	−0.21 ***
	(−0.34–−0.08)	(−0.37–−0.04)	(−0.31–−0.10)	(−0.31–−0.11)
Self-employed	−0.09 †	−0.24 **	−0.11 **	−0.12 **
	(−0.18–0.00)	(−0.39–−0.08)	(−0.19–−0.03)	(−0.20–−0.04)
Unpaid family worker	−0.23 ***	−0.23 †	−0.23 ***	−0.23 ***
	(−0.37–−0.10)	(−0.49–0.03)	(−0.35–−0.11)	(−0.35–−0.11)
≤Elementary	−0.01	0.03	0.02	0.01
	(−0.11–0.08)	(−0.10–0.17)	(−0.07–0.10)	(−0.07–0.10)
≤Middle school	0.03	-0.03	0.02	0.02
	(−0.05–0.12)	(−0.15–0.09)	(−0.05–0.09)	(−0.05–0.09)
≤High school	0.03	−0.08	0.01	0.00
	(−0.07–0.13)	(−0.23–0.06)	(−0.08–0.09)	(−0.08–0.08)
≥College	1.00	1.00	1.00	1.00
	[reference]	[reference]	[reference]	[reference]
Metropolitan	1.00	1.00	1.00	1.00
	[reference]	[reference]	[reference]	[reference]
City	0.03	0.17 ***	0.08 **	0.08 **
	(−0.04–0.10)	(0.08–0.26)	(0.03–0.14)	(0.02–0.13)
Rural	0.03	0.01	0.03	0.04
	(−0.03–0.10)	(−0.10–0.12)	(−0.02–0.09)	(−0.02–0.09)
No chronic disease	1.00	1.00	1.00	1.00
	[reference]	[reference]	[reference]	[reference]
Chronic disease: 1	0.02	0.10 †	0.05	0.05

(0)				
	(−0.06–0.10)	(−0.01–0.20)	(−0.02–0.11)	(−0.02–0.11)
Chronic disease: 2+	0.13 ***	0.16 **	0.14 ***	0.14 ***
	(0.06–0.20)	(0.05–0.26)	(0.08–0.20)	(0.08–0.21)
IADL: 1+ (0)	0.33 ***	0.05	0.24 ***	0.24 ***
	(0.24–0.41)	(−0.08–0.17)	(0.17–0.31)	(0.17–0.31)
ADL: 1+ (0)	0.09 †	0.29 ***	0.14 **	0.13 **
	(−0.01–0.19)	(0.15–0.42)	(0.05–0.22)	(0.05–0.22)
2006 CES-D 10	0.07 ***	0.06 ***	0.06 ***	0.07 ***
	(0.06–0.08)	(0.05–0.08)	(0.06–0.07)	(0.06–0.07)
Household income	-	-	−0.03 †	−0.03 †
			(−0.06–0.00)	(−0.06–0.00)
Household wealth	-	-	0.80 *	0.80 *
			(0.15–1.46)	(0.15–1.44)
Observations	2753	1370	3775	3775

Abbreviations: CES-D 10, Center for Epidemiologic Studies-Depression 10-item Scale; IADL, instrumental activities of daily living; ADL, activities of daily living; CI, confidence interval. *** $p < 0.001$, ** $p < 0.01$, * $p < 0.05$, † $p < 0.1$; Negative Binomial regressions on CES-D 10 score from the 2012 KLoSA (robust standard errors); all adjusted variable, except 2006 CES-D 10 and income level and volatility, are collected from the 2012 KLoSA.
